# Gallbladder Burkitt’s Lymphoma: A Literature Review Including a Case Report in a Child Living with HIV

**DOI:** 10.3390/idr16050078

**Published:** 2024-10-10

**Authors:** Nathalia Lopez Duarte, Ana Paula Silva Bueno, Bárbara Sarni Sanches, Gabriella Alves Ramos, Layanara Albino Batista, Thalita Fernandes de Abreu, Marcelo Gerardin Poirot Land, Cristiane Bedran Milito

**Affiliations:** 1 Faculty of Medicine (FM), Federal University of Rio de Janeiro (UFRJ), Rio de Janeiro 21941590, Rio de Janeiro, Brazil; barbarasarni@ufrj.br (B.S.S.); thalita.abreu@medicina.ufrj.br (T.F.d.A.); land.marcelo@ippmg.ufrj.br (M.G.P.L.); crismilito@medicina.ufrj.br (C.B.M.); 2 Martagão Gesteira Institute of Pediatrics and Childcare (IPPMG), Federal University of Rio de Janeiro (UFRJ), Rio de Janeiro 21941912, Rio de Janeiro, Brazil; apbueno@ippmg.ufrj.br (A.P.S.B.); gabriella.ramos@medicina.uerj.br (G.A.R.); 3 Internal Medicine Department, Central Air Force Hospital (HCA), Rio de Janeiro 20261005, Rio de Janeiro, Brazil; labatista@id.uff.br; 4 National Institute of Science and Technology in Childhood Cancer Biology and Pediatric Oncology (INCT BioOncoPed), Porto Alegre 90035903, Rio Grande do Sul, Brazil; 5 Pediatric Hematology Service, Martagão Gesteira Institute of Pediatrics and Childcare (IPPMG), Federal University of Rio de Janeiro (UFRJ), Rio de Janeiro 21941912, Rio de Janeiro, Brazil; 6 Infectious and Parasitic Diseases Service, Martagão Gesteira Institute of Pediatrics and Childcare (IPPMG), Federal University of Rio de Janeiro (UFRJ), Rio de Janeiro 21941912, Rio de Janeiro, Brazil; 7 Department of Pathology, Faculty of Medicine (FM), Clementino Fraga Filho University Hospital (HUCFF), Federal University of Rio de Janeiro (UFRJ), Rio de Janeiro 21941617, Rio de Janeiro, Brazil

**Keywords:** Burkitt’s lymphoma, gallbladder, malignant lymphoma, gallbladder cancer, HIV, pediatric, case report, Brazil

## Abstract

Malignant lymphoma is an unusual form of gallbladder neoplasm. Almost all these tumors are diffuse large B-cell lymphomas or mucosa-associated lymphoid tissue-type lymphomas. Herein, we present a literature review of gallbladder Burkitt’s lymphoma (BL) cases that includes also an unpublished case in an HIV-infected child, observed by our center. The patient (a five-year-old black female child) attended the Federal Hospital of Lagoa, Rio de Janeiro, Brazil, underwent cholecystectomy, and the postoperative pathological analysis of the gallbladder revealed a diagnosis of BL (EBV-positive). Also, HIV serology was performed and returned positive. She was transferred to the Martagão Gesteira Institute of Pediatrics and Childcare for oncological treatment, dying from sepsis and disease progression about 18 months later. The patient did not undergo ART/cART. Previous cases of gallbladder BL were herein described and analyzed to characterize the clinicopathological features and possible similarities. BL can occur in the gallbladder both in the context of HIV infection and in the pediatric population. A biopsy is mandatory in cases with suggestive findings of lymphoma, and an early diagnosis can change the course of the disease. Furthermore, the case highlights the importance of an early initiation of ART/cART in people living with HIV (PLWH), especially in children.

## 1. Introduction

Malignant gallbladder lymphoma is particularly uncommon [1,2,3,4,5,6]. Most patients diagnosed with this disease are referred for surgery with the diagnostic hypothesis of gallbladder adenocarcinoma or cholecystitis. That is because preoperative diagnosis is exceedingly challenging. Although several reports have documented malignant gallbladder lymphomas, most of these malignancies are diffuse large B-cell lymphomas (DLBCL) or Marginal Zone Lymphomas (MZL) [4,6,7]. Burkitt lymphoma (BL) is a subgroup of high-grade non-Hodgkin’s lymphoma (NHL) characterized by an aggressive clinical course. Patients with BL often present with solid tumors, large lymph nodes, or symptoms resembling acute leukemia, with bone marrow involvement in more than 25% of cases. Most sporadic BL cases occur in the bowel, respiratory tract-associated lymphoid tissue, and gut-associated lymphoid tissue, making the gallbladder an atypical site [8,9]. Only five reports have previously documented gallbladder BL [1,2,8,9,10]. This article aims to conduct a literature review of BL cases published to date, along with reporting a case of BL in a child living with HIV treated at a Brazilian center.

## 2. Literature Review

The first pediatric gallbladder BL case was reported in 1996 by Balonga et al. [10], involving an 11-year-old boy with anemia, abdominal distension, and masses. Imaging revealed extensive abdominal lesions, including in the gallbladder. The diagnosis was based solely on morphology, and chemotherapy reduced the tumor by 75% within eight days. The patient had no significant medical history; follow-up indicated a good prognosis post-chemotherapy.

Hosoda et al. [1] reported the first potential case of primary gallbladder BL in an adult, involving an 83-year-old man with an incidental gallbladder tumor and enlarged periportal lymph nodes in a computed tomography (CT) scan. Despite a history of cerebral artery stenosis and atrial fibrillation, the patient had no abdominal symptoms. Imaging suggested gallbladder adenocarcinoma, but histopathology confirmed BL. After surgery, recurrence occurred, and he was treated with a reduced-dose chemotherapy regimen.

Doherty et al. (2019) [8] reported a 65-year-old man with abdominal pain and distension, initially diagnosed with cholecystitis and pancreatitis. Despite treatment, his symptoms worsened, revealing pancytopenia, hyponatremia, and diffuse abdominal inflammation. A cholecystectomy revealed abnormal findings, and histology confirmed stage IV BL with neoplastic cells in the cerebrospinal fluid (CSF). The patient was subsequently treated with a chemotherapy regimen, including etoposide, vincristine, doxorubicin, prednisone, and rituximab.

Repine et al. (2004) [9] reported a 51-year-old man with a history of gallstones presenting with fever, jaundice, and abdominal discomfort. Imaging revealed a thickened gallbladder wall and a hepatic lesion, initially suspected as an abscess. Biopsies confirmed NHL with gallbladder involvement. The patient was treated with rituximab, systemic, and intrathecal chemotherapy, leading to significant improvement and complete remission.

Finally, Martín et al. (2021) [2] described a case of a 37-year-old HIV-positive male with BL involving the gallbladder, liver, pancreas, peritoneum, and bone marrow. The patient presented with a month-long history of epigastric pain and was classified as stage A2, with a CD4^+^ count of 374 cells. Advanced disease was confirmed by biopsy, with fluorescence in situ hybridization (FISH) revealing the characteristic t(8;14) translocation. He was diagnosed as stage IV-A and required chemotherapy following the diagnosis. There is no information available regarding his use of antiretroviral therapy (ART) or combined antiretroviral therapy (cART). This case represents the first reported instance of gallbladder BL in an HIV-positive individual. 

All these five cases add knowledge to the limited medical literature on adult and pediatric gallbladder BL. Herein, we report the second published case of gallbladder BL in a child, and the second reported case in a patient living with HIV. Notably, this is the first case of a pediatric patient living with HIV diagnosed with this type of lymphoma.

## 3. Case Description

A five-year-old black female from Rio de Janeiro, Brazil, attended the Federal Hospital of Lagoa (HFL) with vomiting, abdominal pain (absence of Murphy’s sign), diarrhea, and jaundice for four days. Previously healthy, she began experiencing symptoms on 06/16/99 and was admitted on 06/20/99 with dehydration and jaundice. Diagnosed with intestinal subocclusion caused by *Ascaris lumbricoides*, she was treated with albendazole but developed severe cholangitis, requiring an emergency cholecystectomy on 07/15/99. Histopathological analysis revealed BL positive for Epstein–Barr Virus (EBV). HIV serology (enzyme-linked immunosorbent assay—ELISA) on 07/28/99 was positive, with a viral load of 200,000 copies/mL, CD4^+^ 2.00%, and CD8^+^ 45.00%. The child was classified as C3 under CDC criteria [11,12]. Vertical transmission (VT) of HIV was confirmed as her mother’s HIV test was also positive, with no history of blood transfusion or sexual abuse. No imaging tests were performed before surgery or for histopathological analysis.

**Figure 1:**
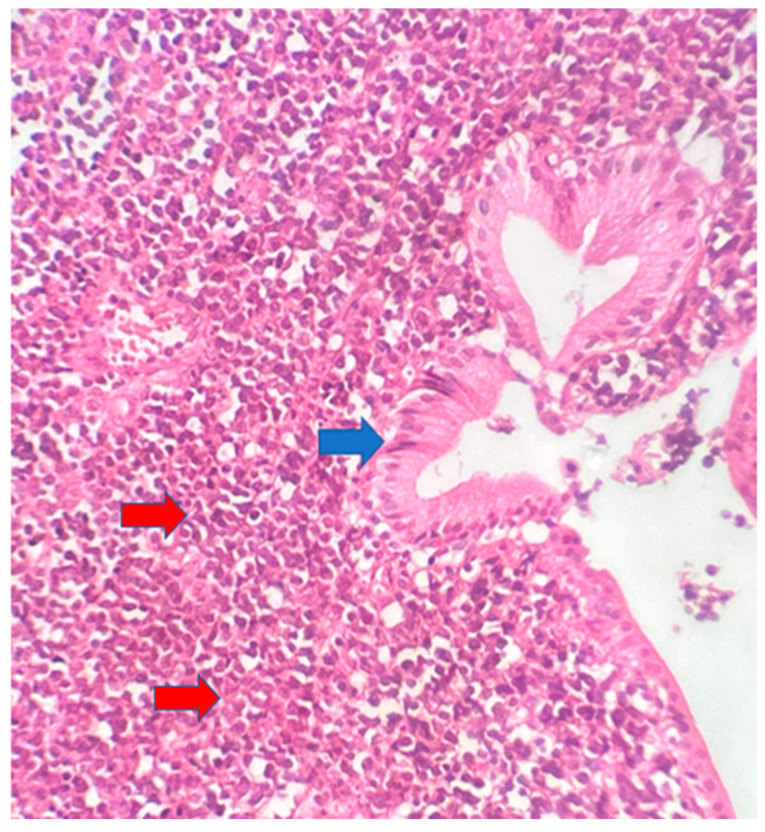
Gallbladder BL. Neoplasm consisting of intermediate-sized cells (red arrows) located in the gallbladder chorion (blue arrow). 10× magnification.

**Figure 2:**
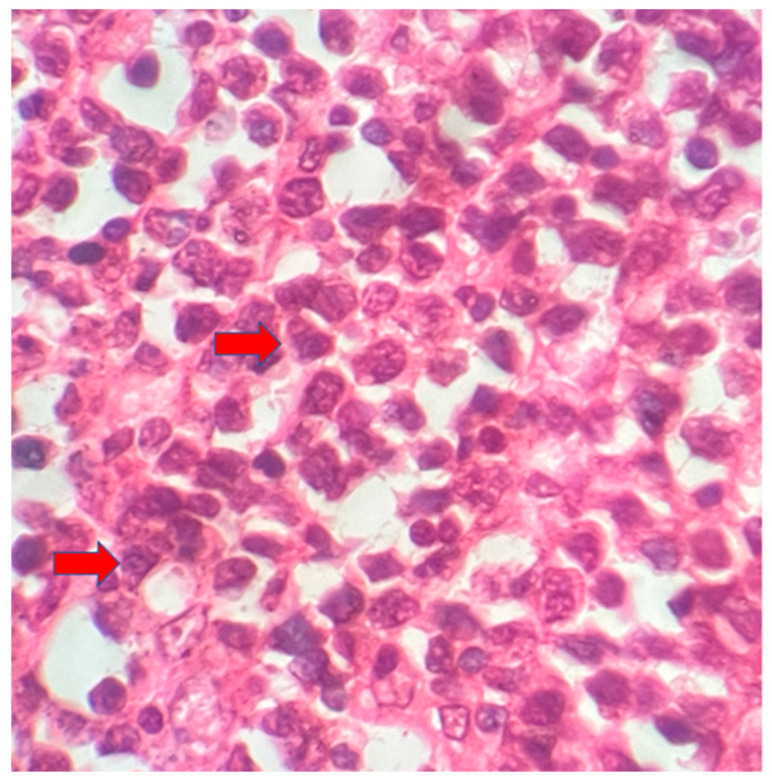
Intermediate-sized cells, with evident nucleoli and diffuse proliferation (red arrows). 40× magnification.

**Figure 3:**
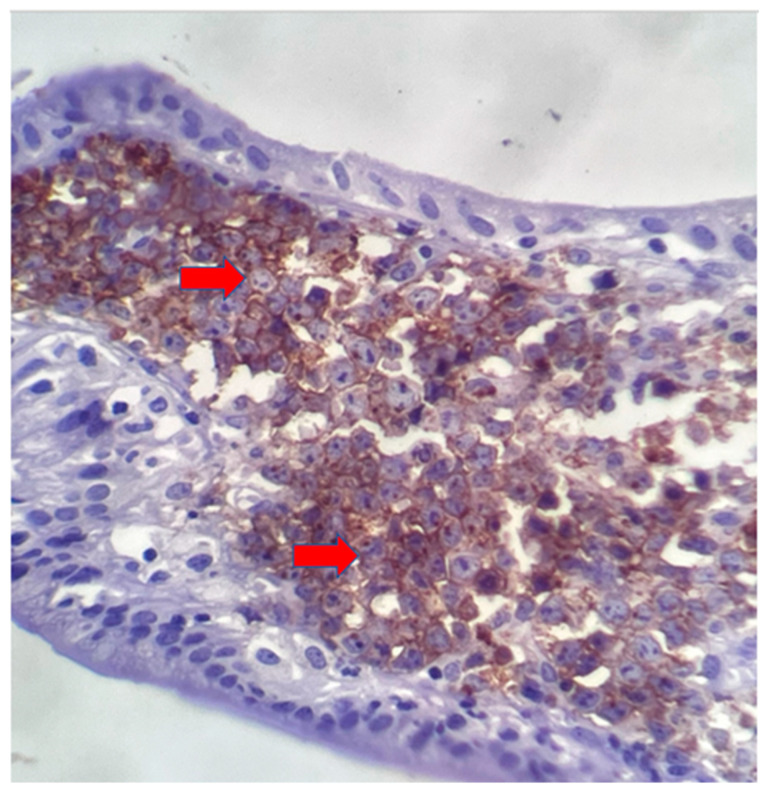
Diffuse immunostaining of cell membrane with anti-CD20 antibody in neoplastic cells (red arrows) and anti-CD10 antibody. 40× magnification.

**Figure 4:**
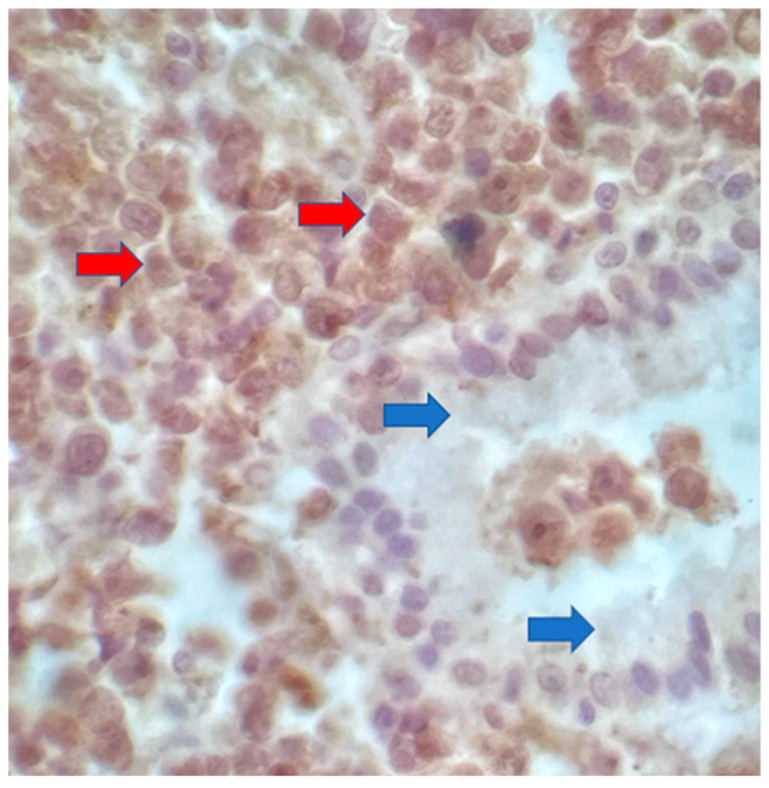
Nuclear immunostaining with anti-Ki67 antibody in all neoplastic cells (red arrows) and negativity in the gallbladder epithelium (blue arrows). 40× magnification.

**Figure 5:**
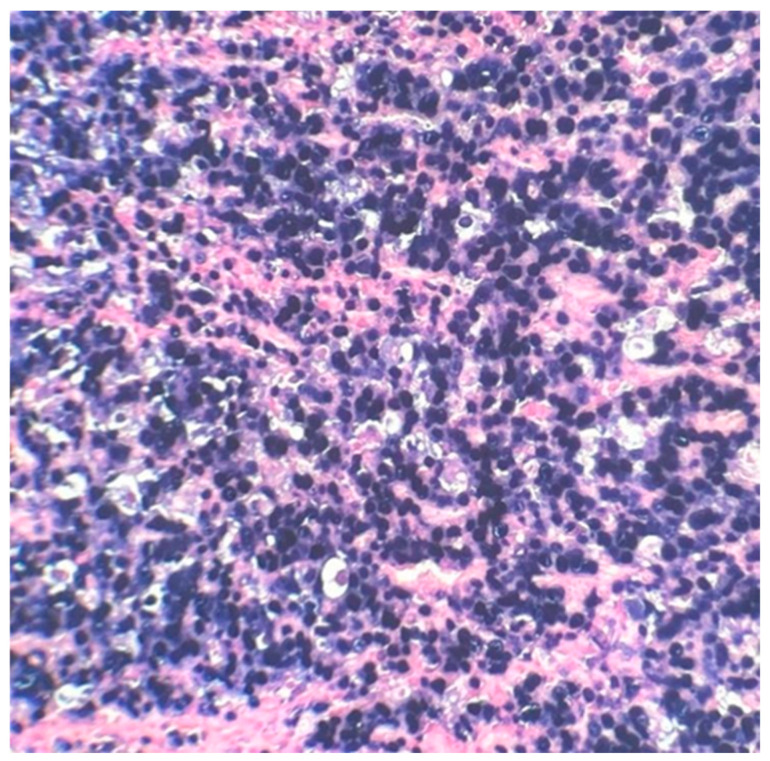
HIS technique showing positivity (blackish nuclei) for the EBER1 probe.

The postoperative histopathological examination of the resected specimen occurred on 07/16/99 at the Department of Pathology of Clementino Fraga Filho University Hospital (HUCFF/UFRJ). The macroscopic examination showed a gallbladder measuring 4 cm × 1.0 cm × 0.9 cm. The serosa was opaque, and the wall thickened and became white, with a firm consistency. The specimen was re-evaluated by a hematopathologist on 08/19/22 (C.B.M), who confirmed the diagnosis of BL through microscopic analysis and immunohistochemical study according to WHO, 2022 [13]. Morphological analysis revealed diffuse proliferation of intermediate-sized cells, with small nucleoli invading and destroying the bladder wall, with a high apoptotic and mitotic index. The neoplastic cells showed positivity for CD20, CD10, Ki67 99%, and EBER by in situ hybridization (EBER1) [13,14]. Combined with the patient’s medical history, the final diagnosis was gallbladder BL. This is an AIDS-defining malignancy (ADM), codified as 2A85.6 (BL) according to ICD-11 MMS [15]. There are five photographs of the sample in question below (Figure 1, Figure 2, Figure 3, Figure 4 and Figure 5).

Transferred to the Martagão Gesteira Institute of Pediatrics and Childcare (IPPMG/UFRJ) on 07/31/99 for oncological treatment, the patient was presented with hemodynamic instability, severe malnutrition, hepatomegaly, and abdominal distension but with no neurological impairment. Initial laboratory tests revealed severe anemia, leukopenia, and thrombocytosis—hemoglobin (Hb) 7.3 g/dL, 3000 leukocytes/mm^3^ (43% neutrophils, 30% lymphocytes, and 17% monocytes) and 485,000/mm^3^ platelets. Bone marrow aspirates on 08/03/99 confirmed L3 Acute Lymphoblastic Leukemia (L3-ALL) by French-American-British Classification (now BL [13]), with a normal CSF. A radiograph of long bones showed lytic lesions in the femur, bilaterally. The patient was then classified as stage IVB lymphoma [16], with a Performance Status (PS) of 4.

The m-BACOD protocol [17] was initiated on 08/04/99, and the NHL-BFM 95 protocol [18] was started after a few weeks due to disease progression. It is essential to mention that the patient was kept off prophylaxis and ART/cART during all treatment periods [19]. Clinical remission was achieved by 09/23/00, but the central nervous system (CNS) relapsed on 10/04/00. A CT scan showed generalized lymphadenopathy, and LDH increased from 220 to 998 U/L. Her clinical condition progressively worsened, and the patient died from sepsis and disease progression on 12/24/00.

## 4. Discussion

Malignant lymphoma of the gallbladder is a rare type of gallbladder malignancy, which encompasses 0.1–0.2% of all gallbladder tumors [1,2,3,4,5]. In prior reports, the majority of them were described to be DLBCL or MZL [4,6]. In all medical literature, just five reports had documented Gallbladder BL [1,2,8,9,10]. The present case report grants some interesting clinical information once it is the second Gallbladder BL described in the pediatric population and the second one in an individual living with HIV as well. It can be observed that most cases of BL involving the gallbladder described in the literature—and reported in this review—occurred in elderly individuals without comorbidities that could be directly related to the tumor onset, such as the presence of gallstones or immunosuppression by HIV [2]. It is known that gastrointestinal symptoms are common in this type of neoplastic involvement [8,9,10]. Interestingly, in one of the cases, the patient did not even present with abdominal pain at the time of diagnosis of the neoplasm [1].

Even though BL responds well to chemotherapy, recurrences are common in patients with this neoplasm due to its aggressive nature [20], and this situation was observed in one of the cases [1]. Due to the localization, cholecystitis and pancreatitis can be critical differential diagnoses for gallbladder BL, with one of the cases initially mistaken for acute acalculous cholecystitis [8] and another for complicated cholecystitis with hepatic abscess [9]. In this context, cholecystectomy is often performed by default, but delay in the institution of chemotherapy can easily result in a poor outcome.

The 11-year-old patient with gallbladder BL had no significant past medical history. However, there is no information regarding HIV testing, which could be a plausible hypothesis for the development of the neoplasm. Unlike our case, the 11-year-old child presented with disseminated abdominal masses (e.g., pancreas, liver, and rectum-sigmoid), and the tumor was not localized solely in the gallbladder. It is important to mention that both gallbladder lesions were similar in size, measuring approximately 4 cm at their largest diameter. Additionally, it is unclear whether the Spanish case was a primary or secondary gallbladder BL due to the absence of other laboratory tests or imaging exams that could contribute to better case elucidation.

Also, as in the new case described in this paper, it is important to emphasize the importance of an early diagnosis to start chemotherapy properly. Since it is a rapidly growing neoplasm, the prognosis for BL depends more on the tumor size than its location, making a quick diagnosis crucial for initiating chemotherapy as soon as possible [20]. In the 1996 case, there was a significant reduction in tumor mass (75%) after eight days of chemotherapy, and the patient’s follow-up indicated a favorable prognosis. Considering that HIV-related lymphomas tend to be systematically more aggressive [21], the Spanish case probably does not affect an HIV-positive patient. Finally, in contrast to the report presented in this article (which was EBV-positive), the Spanish case lacks information regarding the presence of EBV in the biopsy sample. However, it is important to note that this pioneering 1996 case encompasses unique aspects of pediatric BL with atypical localization.

The same can be stated about the case published in 2021 by Martín et al. [2]. Although it was a unique case in the population living with HIV, many important details were not described, such as ART/cART use and whether the tumor was classified as primary or secondary. The patient’s outcome was also not provided. Nevertheless, the aggressive nature of HIV-related neoplasia can be observed, as the individual was only 37 years old at the time of the diagnosis of gallbladder BL. According to the diagnostic criteria of gastrointestinal lymphoma, which was defined by Dawson et al. [22] and Lewin et al. [23], our case is considered a “secondary” gallbladder BL because it included extra-gallbladder lesions, which probably occurred before gallbladder infiltration. Some days after surgery (24 days), the patient underwent an investigation in a quaternary hospital, which diagnosed an L3-ALL. There was no CNS infiltration. Besides this, an x-ray of long bones showed bilateral lytic lesions in the femurs.

Gastrointestinal symptoms (vomiting, abdominal pain, diarrhea, and jaundice) were notable and led the patient to seek medical assistance. It is well known that gallbladder BL can be presented as a localized disease that mimics gallbladder cancer. Ono et al. [5] reported imaging descriptions of malignant lymphomas, showing that high-grade malignant lymphomas can exhibit solid and bulky masses or unconventional wall thickening. Because BL is a highly aggressive and rapidly progressive disease, some extranodal sites are generally involved at the time of diagnosis, as in our case report [20,24].

In children living with HIV (CLWH), the incidence of malignant neoplasms is higher. This substantial increase is related to a high frequency of NHL, particularly B-NHL (like BL), Kaposi’s sarcoma (KS), leiomyosarcoma, and Hodgkin’s lymphoma (HL) [11,21,25,26,27,28].

When lymphomas and HIV occur simultaneously in children with immature immune systems, it can cause serious consequences. HIV-related lymphomas are typically linked to immune dysregulation, as HIV infection causes a depletion of both cellular and humoral immunity [27,29,30,31,32,33,34,35,36]. In this population, lymphomas are often detected at a late stage with the involvement of tissues outside the lymph nodes and usually with a rapid and aggressive progression [21,26,28,37]. It is widely acknowledged that the presence of HIV infection significantly worsens the prognosis for children and adolescents with lymphoma, even with the use of antiretroviral therapy and chemotherapy [38,39,40,41,42,43,44].

The accurate antiretroviral therapy reduced morbidity and mortality in people living with HIV (PLWH) since it inhibits viral replication and restores immunological surveillance [45,46,47,48]. So, starting ART/cART as soon as possible is related to a better recuperation of CD4^+^ T-cell counts and, consequently, to the reduction in problems caused by HIV immunosuppression—such as opportunistic infections and malignant neoplasms [45,46,47,48,49].

In the context of immunosuppression due to HIV infection, ART/cART, in combination with chemotherapy, has the highest priority in BL treatment, even if higher than surgery [1,43,44,45,49]. Unfortunately, the patient died from sepsis and disease progression about 18 months after the lymphoma diagnosis, despite the early beginning of chemotherapy (around 20 days after surgery and histopathological analysis). It is noteworthy that the patient did not undergo ART/cART in any moment during the treatment, although she had been treated in a reference center for HIV and cancer treatment in Brazil (IPPMG/UFRJ). In a recent work published by our research group, which encompasses the referred case, it was observed that patients who achieved complete remission but did not recover CD4^+^ levels had inferior survival rates with higher relapse occurrence and infections related to the rescue protocols instituted [44]. Nowadays, the role of antiretroviral therapy is well-established for a better prognosis of PLWH precisely because it prevents the appearance and recurrence of malignant tumors, especially lymphomas [12,21,43,44,45,49].

Even though the precise preoperative diagnosis of gallbladder malignant lymphoma is difficult, some previous reports [4,5,50,51] have suggested the possibility of an accurate preoperative suspicion of lymphoma centered on imaging analysis. Unfortunately, in the case in question, we did not have access to modern imaging resources for evaluation and comparison with other reports. In addition, the patient had an emergency cholecystectomy due to an initial inaccurate diagnosis (intestinal subocclusion by *Ascaris lumbricoides*), with rapid progression to cholangitis probably because of an obstruction caused by the tumor. Furthermore, the correct mass location in the topography of the gallbladder was not documented in the medical records. As this is a case from 1999, some information was lost, and valuable data for a contemporary case report were not collected. However, this did not harm the diagnosis or the focus of the discussion, once biopsy assessment is the gold standard diagnostic procedure in any lymphoma investigation [1,50,52].

Another important point of our case is the presence of EBV in the sample, confirmed by EBER1 [14]. Transcription of non-polyadenylated RNAs EBER1 and EBER2 is a constant feature of all EBV latent infection patterns and is, therefore, the best marker to demonstrate infection by this oncogenic virus [53,54,55,56]. It is known that EBV is linked to the development of a variety of HIV-related lymphomas [57,58,59], and it is present in 60% of BL cases in PLWH [21]. In sub-Saharan Africa, LB is endemic, and its risk increases both with increasing anti-EBV antibody titers and with HIV infection, for example [59]. Therefore, our finding corroborates the medical literature. However, the role of EBV in malignancies in CLWH across a range of Western countries in the post-cART era is still not fully recognized.

## 5. Conclusions

BL can occur in the gallbladder, especially in the context of immunosuppression—such as caused by HIV infection. To our knowledge, this is the first reported case of gallbladder BL in a pediatric patient living with HIV. It is already known that BL should be contemplated in the differential diagnosis of a gallbladder tumor, and biopsy is mandatory for diagnosis and the early beginning of chemotherapy. Neoplasms may present themselves more aggressively in immunosuppressed patients. In this scenario, an early diagnosis can change the course of the disease. Furthermore, the case highlights the importance of an early initiation of ART/cART in PLWH, especially in children. For this reason, this report is so important, since gallbladder BL should also be considered a differential diagnosis in this population.

## Data Availability

The data presented in this study are available on request from the corresponding author. The data are not publicly available due to ethical restrictions.
